# The key player in the pathogenesis of environmental influence of systemic lupus erythematosus: Aryl hydrocarbon receptor

**DOI:** 10.3389/fimmu.2022.965941

**Published:** 2022-08-30

**Authors:** Jingwen Wu, Tianyi Pang, Ziyuan Lin, Ming Zhao, Hui Jin

**Affiliations:** Department of Dermatology, Hunan Key Laboratory of Medical Epigenomics, The Second Xiangya Hospital, Central South University, Changsha, China

**Keywords:** aryl hydrocarbon receptor (Ah receptor or AhR), systemic lupus erythematosus (SLE), immunology, autoimmune disease (AD), AhR ligands

## Abstract

The aryl hydrocarbon receptor was previously known as an environmental receptor that modulates the cellular response to external environmental changes. In essence, the aryl hydrocarbon receptor is a cytoplasmic receptor and transcription factor that is activated by binding to the corresponding ligands, and they transmit relevant information by binding to DNA, thereby activating the transcription of various genes. Therefore, we can understand the development of certain diseases and discover new therapeutic targets by studying the regulation and function of AhR. Several autoimmune diseases, including systemic lupus erythematosus (SLE), have been connected to AhR in previous studies. SLE is a classic autoimmune disease characterized by multi-organ damage and disruption of immune tolerance. We discuss here the homeostatic regulation of AhR and its ligands among various types of immune cells, pathophysiological roles, in addition to the roles of various related cytokines and signaling pathways in the occurrence and development of SLE.

## 1 Introduction

The aryl hydrocarbon receptor (AhR) was first studied as an environmental toxicant sensor (1). It is a cytoplasmic receptor and transcription factor that relies primarily on ligand activation and subsequently regulates the transcription of many genes by binding to DNA. The AhR pathway is critical in the regulation of innate and adaptive immunity, it’s well established that both AhR agonists and antagonists can affect the immune system. Previous studies have shown that AhR is associated with many autoimmune diseases, including multiple sclerosis, rheumatoid arthritis, inflammatory bowel disease, SLE, and so on (2). By elucidating the role of AhR in autoimmune diseases, researchers can enhance their understanding of the pathogenesis of autoimmune diseases and develop new therapeutic targets. SLE is a classic autoimmune disease characterized by a dysregulation of immune tolerance leading to excessive inflammatory response and multi-organ damage. In this paper, we summarize the homeostatic regulation and pathophysiological roles of AhR and its ligands among various types of immune cells, along with the roles of various related cytokines and signaling pathways in the development of SLE.

## 2 About AhR

### 2.1 Structure and function of AhR

**Figure 1:**
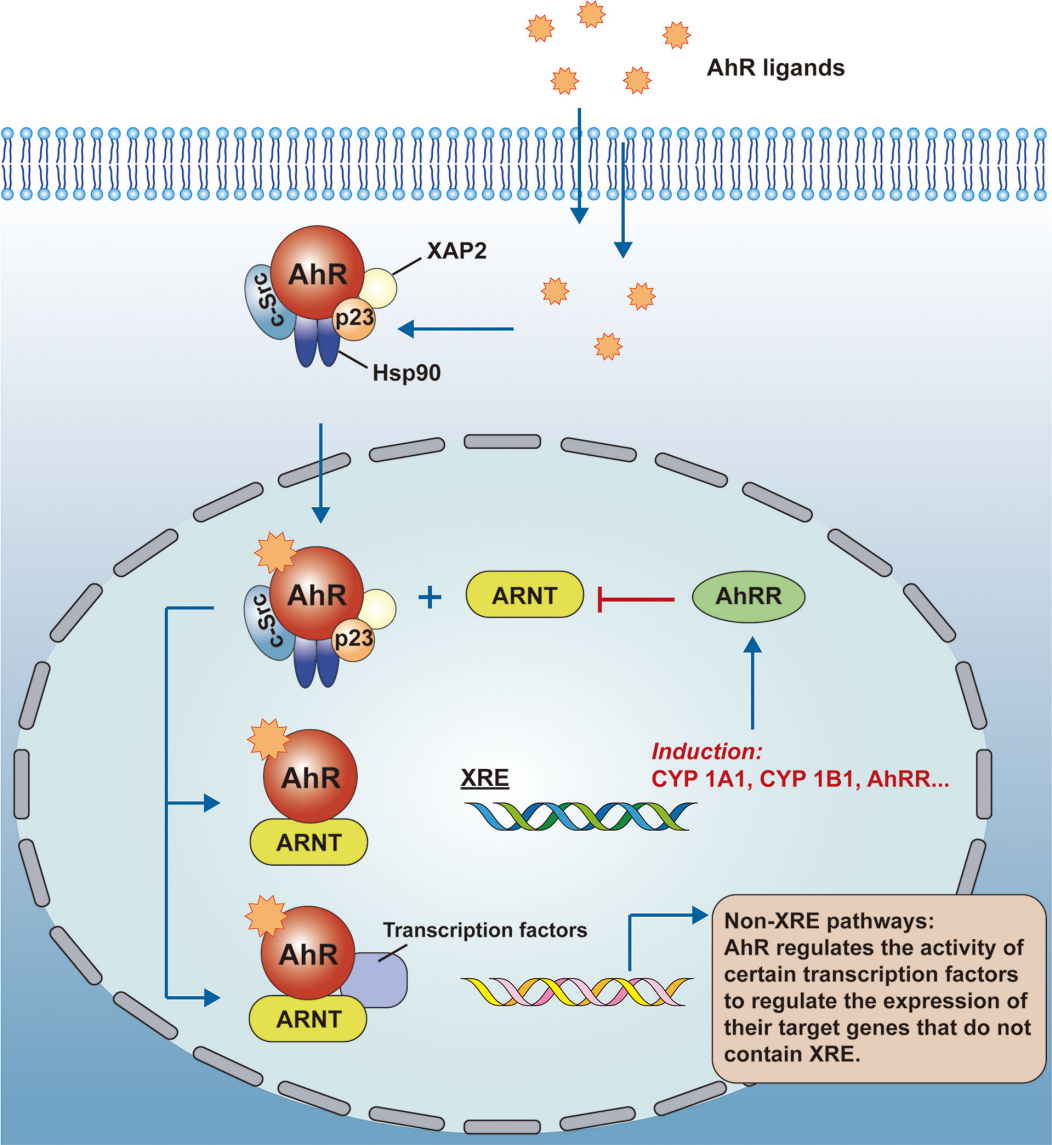
AhR signaling pathway. AhR is a ligand-activated transcription factor and inactive AhR forms a complex with Hsp90, XAP2, c-Src, and p23 in the cytoplasm. As AhR binds to the ligand, conformational changes occur, and they form complexes with the ARNT to enter the nucleus. the genomic regulatory region of AhR target genes contains the XRE, and in the nucleus, besides recognizing the XRE, the AhR-ARNT complex also interacts with other transcriptional regulators to control the expression of multiple target genes. AhR, aryl hydrocarbon receptor; ARNT, aryl hydrocarbon receptor nuclear transport protein; XRE, exogenous response element; CYP, cytochrome P450.

AhR is a class of ligand-dependent activating transcription factors belonging to the basic helix-loop-helix Per-Arnt-Sim (bHLH/PAS) superfamily that regulates the transcriptional expression of genes. The inactive AhR forms a complex with the 90-kDa heat shock protein (Hsp90) (3), the hepatitis B virus X-associated protein 2 (XAP2) (4), the protein tyrosine kinase c-Src (5) and the cochaperone p23 (6) in the cytoplasm. These molecular chaperones help to localize inactive AhR in the cytoplasm, protect it from degradation, and allow ligand binding to AhR to occur easily and consistently. As AhR binds to the ligand, conformational changes occur, and they form complexes with aryl hydrocarbon receptor nuclear transporter protein(ARNT), allowing them to enter the nucleus. The genomic regulatory region of AhR target genes contains specific DNA sequences (5’-TNGCTGG-3’) called dioxin or xenobiotic response elements (XRE). In the nucleus, the AhR-ARNT complex recognizes XRE, it controls the expression of multiple target genes through interaction with other transcription factors, such as CYP1A1, CYP1B1, and aryl hydrocarbon receptor repressor (AhRR) (7). AhR also controls gene expression through non-XRE DNA-responsive elements by interacting with other transcription factors in a similar way, such as nuclear factor-κB (NF-κB), c-Maf, retinoic acid receptor, estrogen receptor (ER), and retinoblastoma (Rb). AhR acts as a regulator of their activity, and with it the expression of their target genes (8). In conclusion, AhR promotes the expression of genes in regulatory regions, in addition to XRE, by recruiting new DNA sequences or by interacting with certain proteins (See **Figure 1** on the AhR signaling pathway for details).

### 2.2 AhR is affected by multiple endogenous and exogenous factors

AhR is known as an environmental receptor and its ligands are very diverse that can be broadly classified into exogenous and endogenous according to their sources. Exogenous ligands are mainly from environmental toxins, including dioxins (9), tetrachlorodibenzo-p-dioxin(TCDD), polychlorinated biphenyl (PCB), polycyclic aromatic hydrocarbon (PAHs) (10), etc. The binding of PAHs to AhR/ARNT induces the expression of several CYPs that convert PAHs into genotoxic pro-electron derivatives. Foods are also rich in natural ligands (e.g., polyphenols, resveratrol (11), quercetin (12), flavonoids (13), etc.) and also include drugs such as cyproheptadine (14), leflunomide (15), and omeprazole (16).

The endogenous ligands mainly include tryptophan derivatives, such as FICZ (17), indole-3-carbinol (I3C) (18), indole-3-acetic acid (IAA) (19) and 2-(1’H-indole-3’-carbonyl)-thiazole-4-carboxylic acid methyl ester (ITE) (20). Also, there are prostaglandins (PGB3, PGD3, PGF3alpha, PGG2, PGH1, PGH2) (21), indocyanine and indigo (22), bilirubin and biliverdin (23), etc. Tryptophan and its derivatives provide a large number of ligands for AhR, and we are familiar with kynurenine (Kyn) as an agonist of AhR (24), while AhR regulates the expression and activation of indoleamine 2,3-dioxygenase (IDO), tryptophan 2,3-dioxygenase (TDO2), kynurenase (KYNU), etc. These enzymes regulate the metabolism of Kyn, which precisely forms a feedback loop. Among them, FICZ, fully known as 6-formylindolo[3,2-b]carbazole, is the endogenous agonist that binds most tightly to AhR (25). See **Table 1** on the classification of factors that can affect AhR for details. Notably, Kyn and the entire tryptophan family are regulated by the mechanistic target of rapamycin (mTOR), a serine/threonine kinase. mTOR is named after rapamycin, an antifungal macrolide antibiotic. mTOR activation and its pro-inflammatory effects profoundly influence the development of SLE, and there is an extensive link between mTOR and AhR signaling pathway, which will be described in 5.5.

**Table 1:** Endogenous and exogenous factors that can affect AhR.

Factors	Origin	Compounds	References
Exogenous	Environmental toxins	Dioxins (e.g. Tetrachlorodibenzo-p-dioxin,TCDD)	(1, 9)
		Polycyclic aromatic hydrocarbons (PAHs)	(10)
		Polychlorinated biphenyl (PCB)	(10)
	Ultraviolet	–	(26)
	Dietary	5-hydroxyindole-3-acetic acid (5-HIAA)	(27)
		Resveratrol	(11)
		Quercetin	(12)
		Flavonoids	(13)
		3,3’-diindolylmethane (DIM)	(18)
		Curcumin	(28)
		Baicalein	(29)
		Norisoboldine	(30)
	Drugs	Cyproheptadine	(14)
		Leflunomide	(15)
		Omeprazole	(16)
Endogenous -	Tryptophan derivatives	6-formylindolo[3,2-b]carbazole (FICZ)	(17)
		Kynurenine (Kyn)	(24)
		lndole-3-carbinol (I3C)	(18)
		Indole-3-acetic acid (IAA)	(19)
		2-(1’H-indole-3’-carbonyl)-thiazole-4-carboxylic acid methyl ester (ITE)	(20)
	Prostaglandin	PGB3, PGD3, PGF3alpha, PGG2, PGH1,PGH2	(21)
	Plant enzymes	Indocyanine and indigo	(22)
	Hemoglobin metabolism	Bilirubin and biliverdin	(23)
	Gut microbiota	Lactobacillus and Bifidobacterium	(31)
		Lactobacillus bulgaricus	(32)

## 3 Relevance of AhR to immune cells

### 3.1 AhR in dendritic cells

Dendritic cells (DCs) are the major antigen-presenting cells in human body. Monocytes enter tissues and differentiate into macrophages or dendritic cells. This differentiation process is regulated by several transcription factors. In the presence of AhR activation, monocyte-derived DCs differentiation is promoted through induction of BLIMP-1 (33). However, AhR is controversial in inducing the differentiation of DCs, which have several subsets: conventional DCs (cDCs) and plasmacytoid dendritic cells (pDCs) (34). TCDD can induce AhR expression and activation in DCs to enhance DCs differentiation (35), these experiments also demonstrated that the AhR pathway functioned as an important signaling pathway for the activation of indoleamine 2,3-dioxygenase 1(IDO1) and indoleamine 2,3-dioxygenase 2(IDO2) expression. IDO1 and tryptophan 2,3-dioxygenase-2 (TDO2) promote the production of the endogenous AhR ligand Kyn and generate DCs. In another study, in the presence of lipopolysaccharide or CpG, AhR negatively regulates DCs-mediated immune responses through a Kyn-dependent mechanism, which in turn affects the differentiation of primary T cells to regulatory T (Treg) cells and T helper 17 (Th17) cells (36).

AhR can also affect the antigen-presenting function of DCs (37). Jaishree Bankoti et al. reported that the AhR ligand TCDD decreased the expression of CD11c on the surface of bone marrow-derived dendritic cells (BMDCs) but increased the levels of MHC II and CD86 on BMDCs. The binding of CD86 molecules on DCs to CTLA-4 on T cells can impair T cell responses (38). As a consequence of AhR activation, CTLA-4 can be expressed on T cells, which binds tightly to CD86 on DCs, impairing T cell responses (39). As such, the attachment of CD86 on DCs to CTLA-4 on T cells, which is triggered by AhR activation, may contribute to immunosuppression. Comparatively, FICZ and ITE induced phenotypic changes similar to those seen in TCDD in BMDCs, indicating that DCs lack a particular response to AhR ligands.

### 3.2 AhR in macrophages

Macrophages are important components of intrinsic immunity, with roles such as phagocytosis and killing of pathogens, participation in the inflammatory response, and involvement in the regulation of adaptive immunity. Macrophages produce pro-inflammatory cytokines, such as IL-6, IL-12, and TNF-α, which activate T cells and induce their differentiation (40). Researchers have demonstrated that AhR suppresses the differentiation of monocytes and bone marrow-derived macrophages (33, 41). There are two subtypes of macrophages: M1 and M2 macrophages (42). FICZ can affect the balance between M1 and M2 macrophages by altering macrophage polarization through the activation of AhR (43). Yang et al. further demonstrated that AhR activation can reduce macrophage differentiation through the AhR-miR-142a-IRF1/HIF-1α pathway to reduce M1 macrophage polarization and promote M2 macrophage polarization (44).

In addition to affecting differentiation, AhR has other regulatory effects on macrophages, as Shinde et al. reported that macrophages exposed to apoptotic cells could activate AhR on their surface and promote the production of the immunosuppressive cytokine IL-10, thereby limiting the development of SLE in mice (45). After treatment with the endogenous AhR ligand FICZ and the exogenous ligand benzo-alpha-picryl, the abundance, ubiquitination, and phosphorylation of a variety of proteins in macrophages can be affected (46).

### 3.3 AhR in mast cells

Mast cells (MCs) contribute to the regulation of mucosal immune and allergic responses. Mast cells can be activated and release inflammatory mediators and cytokines by a variety of stimuli and have a surface that expresses a large number of IgE Fc receptors, which when bound to IgE and cross-linked to antigens can lead to a variety of pathophysiological events.

AhR is an important factor in mast cell activation. Exposure to the AhR ligand FICZ leads to activity reduction of the SHP-2 gene, enhancing the activation of mast cells by IgE (47, 48). Notably, different doses of FICZ stimulation lead to different changes in MCs. Sibilano R et al. reported that repeated exposure to FICZ inhibited MCs’ degranulation, the mechanism is that the release of histamine is FICZ dose-dependent, but is attenuated by repeated activation of AhR (49). Kyn can also activate mast cells by activating AhR to promote a series of reactions such as degranulation and leukotriene release (50, 51). Meanwhile, although AhR can regulate the function of MCs, experiments by Caroline Pilz et al. support that AhR does not have much role in regulating the number of MCs in mice (52).

The intricate relationship between MCs and T cells can serve as a fulcrum for regulating the balance between various types of T cells. On the other hand, in the presence of activated MCs, the Tregs/Th17 balance is tilted towards Th17 accompanied by a decrease in IL-6 abundance and a lack of Th1/Th2 cytokines (53), and this also study showed that MCs, as a source of inflammatory mediators, could counteract the suppressive effect of Treg cells on effector T cell-mediated immunity.

### 3.4 AhR in natural killer cells

Natural killer cells (NK cells) are derived from bone marrow lymphoid stem cells and, upon maturation, settle mainly in the spleen and liver (54). NK cells are innate lymphocytes capable of producing inflammatory cytokines that nonspecifically kill tumor cells and virally infected cells. In terms of NK cell development and differentiation, treatment of human embryonic stem cells (hESCs) with the AhR antagonist StemReginin-1(SR-1) and the AhR agonist TCDD, respectively, revealed that SR-1 increased the differentiation of NK cells while TCDD inhibited the development of them (55).

AhR has many important effects on NK cell function. Petr Bachleda et al. reasonably hypothesized that the effect of resveratrol on NK cytotoxicity is related to the partial agonistic activity of resveratrol on AhR (56), this hypothesis is based on the fact that resveratrol enhances perforin expression and NK cell cytotoxicity through the NKG2D-dependent pathway (57). Kyn can also enhance NK cell cytotoxicity by activating AhR (58). During infection, NK cells are one of the main sources of IL-10, and AhR activation is required for maximal IL-10 production (59). In hematologic cancer, AhR activation using FICZ not only improves the ability of NK cells to produce IFN-γ and cytolytic activity, but also enhances the ability of NK cells to inhibit cancer growth in an AhR-dependent manner (60).

### 3.5 AhR in T cells

#### 3.5.1 AhR and CD4- CD8 -T (DN T) cells

The role between AhR and DN T has received less attention previously, but there is also a link between the two. Maturation of T cells from DN T to TCRαβ+ or TCRγδ+ cells occurs in the thymus, and the frequency of fetal DN TCRγδ+ cells is higher after activation of AhR using TCDD, suggesting that TCDD plays an important role in the differentiation and lineage commitment of DN T cells (61). Kyn stimulates the activation of mTOR complex 1 (mTORC1) in DN T cells of SLE patients (62).

#### 3.5.2 AhR in T helper 1 (Th1) cells, T helper 2 (Th2) cells

It is believed that Th1 cells and Th2 cells antagonize each other in mediating immunity: Th1 mediates cellular immunity and promotes inflammation; Th2 cells mediate humoral immunity and inhibit inflammation (63).

AhR and Th1 cells are of interest in the body’s response to certain bacterial, viral, and parasitic infections. Th1-related immunity is initiated *in vivo* after Trypanosoma cruzi infection, and some AhR ligands have therapeutic effects, but Laura Fernanda Ambrosio et al. found that there is a threshold for AhR activation in this process. The use of higher affinity ligands (above threshold activation) limits CD8+ T cell development and promotes Treg cell development, enabling an earlier suppression of Th1-type responses. Conversely, the use of low-affinity ligands (below-threshold activation) promotes early inflammatory Th1-type responses, thereby limiting parasite replication. Thus, AhR induces multiple regulatory pathways that ultimately affect parasite replication and infection outcomes (64). Eliseu et al. reported that both AhR agonist Kyn and AhR antagonist CH223191 decreased the number of Th1 cells in a mice pulmonary fungal infection model, while agonist FICZ resulted in the expansion of all CD4+ T cell subsets (Th1, Th2, Th17, Th22, and Treg) (65). In addition, AhR-deficient mice displayed a decrease in Th1, Th22, and other immune cells in their lungs (66).

AhR is important as an environmental sensor in regulating the exposure of the organism to exogenous substances, a process that is closely related to Th2. V J Schulz et al. found that activation of AhR with TCDD can suppress Th2-mediated allergic responses by inducing differentiation of CD4+ T cell subpopulations to Treg cells (67). Exposure to ambient particulate matter (PM) (PAHs, dioxins, and heavy metals are the main components) can promote or exacerbate allergic responses by activating AhR in Th2 (68, 69) and enhanced Th2-mediated allergic response enhances the sensitivity of patients to allergens (70).

The imbalance between Th1/Th2 cells has been considered one of the pathogenesis of autoimmune diseases. AhR can regulate the Th1/Th2 balance by activating Th0 cells, and a synthetic anti-allergy agent M50367 (as AhR ligand) exerts anti-allergic effects by suppressing the differentiation of Th0 cells into Th2 cells *in vitro*, tilting the Th1/Th2 balance in favor of Th1 (71). Kakutani et al. demonstrated that low-dose, prolonged exposure to TCDD resulted in a dose-dependent significant increase in Th1 and Th2 lymphocyte responses, similarly shifting the Th1/Th2 balance towards Th1 (72).

#### 3.5.3 AhR in Th17 and Treg cells

Th17 and Treg cells share a common precursor CD4+ T cell, and both require tumor growth factor (TGF)-β to induce differentiation. Nevertheless, their functions are opposite: Th17 cells tend to exacerbate autoimmune and inflammatory responses, while Treg cells suppress autoimmunity and maintain immune homeostasis (73).

AhR is expressed at the highest levels in human Th17 cells, and substantial evidence shows that AhR is necessary for IL-22 production and Th17 differentiation. It is important to note that AhR is unnecessary for the initial differentiation of Th17 cells, but is necessary to promote their expansion and production of IL-22 (74, 75). Many AhR ligands exhibit inhibitory effects on Th17 development and differentiation, such as TCDD, Kyn, I3C and diindolylmethane (DIM) (76–78). Some ligands block Th17-induced immune responses, such as curcumin and naphthoflavone (79, 80). It is thoroughly studied that FICZ can promote the differentiation of Th17 cells (81). Notably, Kyn has a double role, in addition to suppressing differentiation, it also promotes the differentiation of Th17 cells in a ligand-dependent manner, this may be related to the expression of the System L transporter on cells (82). I3C, although recognized to inhibit Th17 cell production and promote Treg production, increased Th17 cell expression in small intestinal cells in the non-obese diabetic (NOD) mice (83). Moreover, though AhR is unnecessary for Th17 differentiation, its activation promotes further function and differentiation of Th17, leading to the development of various autoimmune diseases dependent on Th17 cells.

Transcription factor Foxp3 drives the differentiation and function of Treg cells. AhR not only induced FoxP3 expression directly (76), but also stabilized FoxP3 expression by increasing enhancer activity (84). TCDD, I3C, DIM, ITE, and Kyn, as well as the recent discovery of baicalein and norisoboldine (NOR) (35, 74, 76, 85, 86), have been shown to promote Treg differentiation.

**Figure 2:**
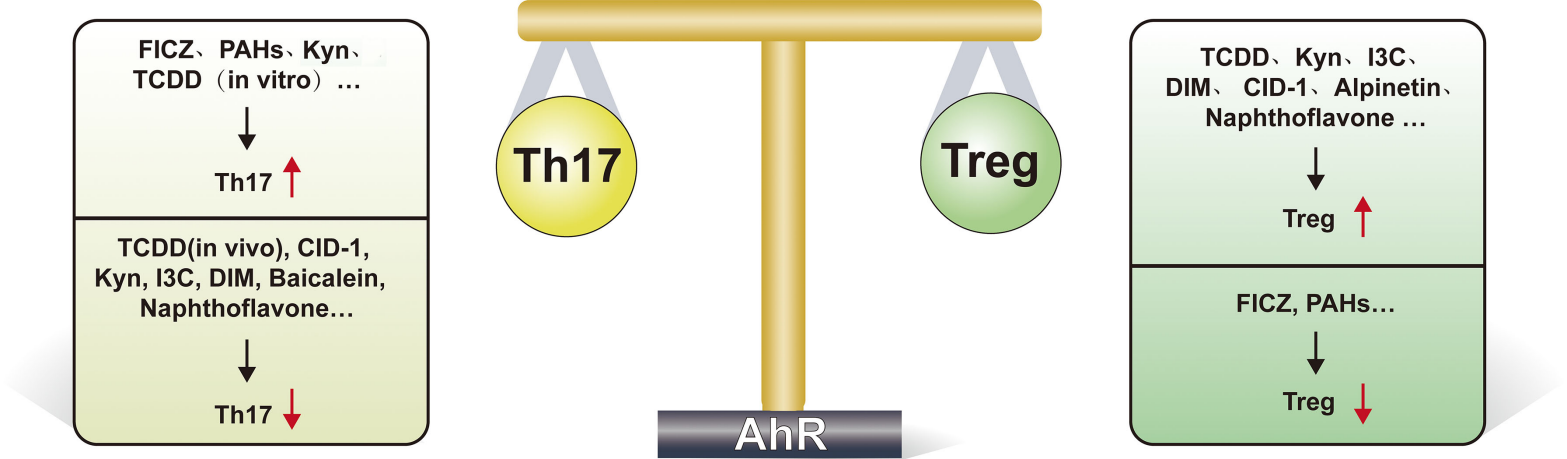
AhR regulates Th17/Treg cell homeostasis delicately. This figure highlights the important role of AhR in regulating Th17/Treg cell homeostasis. As shown, many ligands(such as TCDD, Kyn, I3C, DIM, CID-1, alpinetin and naphthoflavone) promote Treg differentiation after activation of AhR, while FICZ, PAHs suppress Treg differentiation. FICZ, PAHs, Kyn and TCDD (when acting *in vitro*) can promote Th17 cell differentiation, while TCDD (when acting *in vivo*), CID-1, Kyn, I3C, DIM, baicalein and naphthoflavone suppress Th17 differentiation. FICZ, 6-formylindolo[3,2-b]carbazole; Kyn, kynurenine; I3C, indole-3-carbinol; DIM, diindolylmethane; TCDD, Tetrachlorodibenzo-p-dioxin; PAH, Polycyclic aromatic hydrocarbons; CID-1, Cinnamtannin D1.

The regulation of the Treg/Th17 homeostasis by most ligands is unidirectional, that is, promoting one side and inhibiting the other (see **Figure 2** for details). However, there are still some interesting results. TCDD activates AhR and induces the conversion of CD4+ Foxp3 T cells to functional Treg cells (76, 84). Interestingly, in the regulation of Th17, available evidence suggests that *in vitro* use of TCDD activates AhR to promote Th17 differentiation (87), but *in vivo*, TCDD leads to a decrease in Th17 numbers and suppresses experimental autoimmune encephalomyelitis (EAE) occurrence (76), this interesting phenomenon may be due to the fact that humans express the lower-affinity AhR (88), and the difference in affinity of AhR affects the amount of IL-17 and IL-22 produced by Th17 cells, and the number of these cytokines is significantly lower in the mouse model expressing the low affinity AhR. This different role of AhR *in vivo* and *in vitro* fully reflects the complexity of its pathway (87). The use of the AhR antagonist resveratrol interferes with the differentiation of both Treg and Th17 (76). Endogenous ligand FICZ promotes Th17 differentiation (74, 89), and its effect on Treg is controversial; some studies indicate that FICZ reduces the number of Treg cells (89), but more evidence suggests that FICZ promotes Treg production (66, 90). It was also shown that the tryptophan breakdown product Kyn (91) and the flavonoid compound alpinetin (92) increased Treg production but had no effect on Th17 cell production.

#### 3.5.4 AhR and T helper 22 (Th22) cells

Th22 cells belong to the CD4+ T cell subpopulation and are important components of anti-microbial resistance in the mucosa. Th22 has an important impact on a variety of autoimmune diseases including Hashimoto’s thyroiditis (93), rheumatoid arthritis (94), Crohn’s disease (95), and leukoaraiosis (96). AhR deficiency suppresses Th22 expansion, and in a mouse model of pulmonary fungal disease, AhR also regulates the number of Th17 and Treg cells associated with Th22 (66). Th22 secretes IL-22 and AhR is the main transcription factor regulating IL-22 transcription (97), blocking AhR with the antagonist CH223191 decreases IL-22 production (65).

#### 3.5.5 AhR and type 1 regulatory T (Tr1) cells

Tr1 cells are CD4+ regulatory T cells that suppress inflammation and autoimmunity by secreting the immunosuppressive cytokine IL-10. In human peripheral blood mononuclear cells (PBMCs), AhR promotes Tr1 cell differentiation and IL-10 production *via* granzyme B (84). IL-27 can promote the growth and differentiation of Tr1 cells (98). DCs secrete IL-27 after being stimulated by anti-CD3. IL-27R initiates the synthesis of AhR and the transcription factor c-maf, which interact to further expand Tr1 cells *in vivo*(99, 100). According to Gandhi R et al., activation of AhR using TCDD causes differentiation of Tr1 cells *in vitro*(84). Mascanfroni et al. demonstrated that AhR promotes hypoxia-inducible factor 1-α (HIF1-α) degradation and regulates Tr1 cell metabolism during the late stages of differentiation (101).

### 3.6 AhR and B cells

IL-4 regulates AhR expression and activation in B cells, according to Tanaka G et al. (102). Bharat Vaidyanathan et al. performed several experiments to clarify the role of AhR in B cell antibody class switching, differentiation into plasma cells, and generation of memory B cells. The impact of AhR in several developmental processes, AhR may therefore be a novel molecular target for the regulation of B cell immune response (103). It has been more thoroughly studied that the AhR agonist TCDD causes B cells to differentiate in a manner that suppresses antibody production, by reducing the expression of two transcription factors, EBF1 and PAX5 (104, 105). Bach2 is a transcriptional target of AhR in mouse B cell lines, and AhR-mediated transcriptional regulation of Bach2 is a mechanism through which TCDD suppresses B cells (106).

Regulatory B cells (Bregs) are capable of regulating immunity and inflammation, and AhR can promote the differentiation and function of Bregs (107).B10 cells are a specific subpopulation of Bregs, and PGE2 may induce B10 cell expansion through the AhR signaling pathway. Rosser EC et al. found that butyrate supplementation activated AhR by increasing levels of the serotonin-derived metabolite 5-hydoxyindoleacetic acid (5-HIAA), suppressing rheumatoid arthritis in a Bregs-dependent manner (27).

In particular, the numbers and differentiation of T cell subsets have been shown to be affected by cytokines produced by B-cell subsets. In this way, AhR appears to play a multifaceted regulatory role in immune cell differentiation, involving both direct and indirect interactions between immune cells.

The roles of AhR and its ligands in various types of immune cells are shown in **Table 2**.

**Table 2:** Effect of AhR ligand activation on immune cells.

Immune cells	Relevant AhR ligand types	Post-excitation effect	References
Macrophages	FICZ	Affects the abundance, ubiquitination, and phosphorylation of multiple proteins in macrophages	(46)
Dendritic cells	TCDD	Promotes differentiation	(35)
	Kyn	Promotes/suppresses differentiation	(36)
	I3C	Suppresses differentiation	(108)
	TCDD, FICZ, ITE	Leads to immunosuppression	(38)
Mast cells	FICZ	Promotes differentiation at low doses, suppresses differentiation at high doses	(47–49)
	Kyn	Promotes activation	(50)
	TCDD	Promotes Treg cells differentiation	(67)
Natural killer cells	TCDD	Suppresses differentiation	(55)
	StemReginin-1	Promotes differentiation	
	Resveratrol, Kyn	Enhances cytotoxicity of NK cells	(56, 58)
	FICZ	Inhibits cancer growth	(60)
DN T	TCDD	Influences the differentiation and lineage commitment of DN T	(61)
	Kyn	Stimulates mTORC1	(62)
Th1	TCDD	Increases Th1 cytokine production	(109)
		Shifts the Th1/Th2 equilibrium toward Th1	(72)
Th17	FICZ, PAHs. Kyn	Promotes differentiation	(74, 81, 82)
	TCDD	Promotes differentiation *in vitro*, suppresses differentiation *in vivo*	(38, 51)
	CID-1, Kyn, I3C, DIM, Naphthoflavone, Baicalein	Suppresses differentiation	(76–78, 86)
	Curcumin, Naphthoflavone	Blocks Th17-induced immune responses	(79, 80)
Treg	FICZ	Promotes/suppresses generation	(89, 90)
	Resveratrol	Interferes differentiation	(76)
	TCDD, CID-1, Kyn, I3C, DIM, Resveratrol, Alpinetin, Baicalein, Norisoboldine	Promotes differentiation	(35, 74, 76, 85, 86)
	PAHs	Suppresses differentiation	(110)
Th22	–	Promotes differentiation	–
Tr1	TCDD	Promotes differentiation	(84)
B cells	FICZ	Regulates B cell differentiation	(111)
	TCDD	Suppresses differentiation	(104)

FICZ, 6-formylindolo[3,2-b]carbazole; TCDD, Tetrachlorodibenzo-p-dioxin; Kyn, kynurenine; I3C, indole-3-carbinol; CID-1, Cinnamtannin D1; DIM, diindolylmethane; DN T, CD4- CD8- double-negative T cell; Th17, T helper cell 17; Tr1, type 1 regulatory T cells; Treg, regulatory T cells; Th22, T helper cell 22.

## 4 The role of AhR in autoimmunity and autoimmune diseases

The incidence and prevalence of autoimmune diseases continue to increase and current studies have shown that genetic susceptibility accounts for approximately 30% of all autoimmune diseases, the remaining 70% is due to environmental factors (112), which are closely related to AhR. According to the previous section, we have learned that AhR is essential for immune function, and its role in autoimmunity is mainly achieved by regulating the differentiation and function of multiple immune cells, thus affecting autoimmune diseases. In particular, AhR is essential for maintaining the balance between Th17 and Treg cells, which plays a major role in autoimmune diseases (39), and also controls the differentiation and activity of specific T-cell subsets.

In the studies available to date, most of the evidence suggest that AhR activation suppresses inflammatory responses and alleviates autoimmune diseases. Multiple sclerosis (MS) is a demyelinating disease of the central nervous system, in which AhR also plays an active role. In a mouse model of EAE, microbial metabolites limit the pathogenic activity of microglia and astrocytes and suppress CNS inflammation through an AhR-mediated pathway (113). AhR can also be used to monitor disease activity, Rothhammer V et al. detected that AhR agonists are dynamically regulated during MS, and different levels of AhR agonists in serum have an important impact on the progression and prognosis of patients (114).

AhR is also an important regulator of systemic autoimmune diseases. Type 1 diabetes mellitus(T1D) develops due to severe destruction of pancreatic β-cells caused by islet cell autoantigen targeting. AhR activation attenuates the autoimmune response during the development of T1D (115). The most commonly used mouse model for T1D is the NOD mouse, which develops spontaneous disease similar to that of humans (116). Both Kerkvliet NI and Ehrlich AK et al. demonstrated effective suppression of T1D-related symptoms after treatment of NOD mice with AhR ligands (117, 118). Rheumatoid arthritis(RA) affects approximately 1% of the population and is characterized by chronic inflammation of the synovium and joint destruction. Its pathogenesis is unclear, and a variety of factors such as genetics, infection, smoking and environmental pollution may exacerbate the symptoms of RA (119). For example, smoking is one of the major risk factors. Cigarette smoke contains many AhR ligands, 3-MC, BaP and TCDD upregulate IL-1β mRNA in human-like synovial cell lines, and the AhR antagonist α-naphthoflavone inhibits the action of 3-MC (120). AhR has a critical impact on the development of Th17 cells, IL-6 induces Th17 cells and contributes to RA development. Nguyen NT et al. showed that the reduction of Th17 cells in a mouse model of RA by blocking IL-6 may be partially dependent on the inhibition of AhR expression and that AhR antagonists are therefore a promising therapeutic agent for RA (121). Taking all these evidence together, it could be said that AhR is indispensable for RA development.

The main character of this article, SLE, is also a very representative systemic autoimmune disease, and more details about AhR and SLE will be discussed below.

## 5 Role of AhR in the development of SLE

### 5.1 Environmental factors influence the development of SLE through AhR pathways

#### 5.1.1 Gut microbiota affects SLE through AhR pathways

In recent years, the gut microbiota has been studied more intensively and can influence food and drug metabolism, human consciousness and behavior, the pregnancy process, and many other aspects. A growing number of studies have shown multiple associations of gut microbiota with AhR and autoimmune diseases.

On the one hand, the presence of dysregulated gut microbiota in many autoimmune diseases is associated with the activation of AhR signaling (122), and the regulation of gut flora by AhR is in turn modulated by multiple factors, which have been demonstrated in autoimmune diseases such as inflammatory bowel disease (IBD) (122–125). On the other hand, the gut microbiota itself can metabolize or produce some AhR ligands that affect the development of autoimmune responses (126–128).

There is no evidence that the gut microbiota plays an important role in the development of SLE through AhR pathways, and Adriana Cuervo’s group first demonstrated the presence of gut microbial dysbiosis in SLE patients (129), and it has even been suggested that new biomarkers of SLE may be found in the human microbiota (130). In a dietary investigation of SLE patients, the altered abundance of Lactobacillus and Bifidobacterium in the gut was directly correlated with the intake of flavonoid-rich apples and oranges. Some of these strains have immunomodulatory effects and promote Tregs/Th17 differentiation (31). Tryptophan has been reported to be catabolized differently in SLE patients (131). Brown J et al. demonstrated that intestinal flora promoted the production of tryptophan metabolites, including Kyn, which enhanced T cell activation in lupus mice (132). Feeding a high tryptophan diet (including an increase in Kyn) to lupus-prone TC mice resulted in dysbiosis of their intestinal flora and stimulated autoantibody production and produced lupus-like disease when their feces were transferred to germ-free kindred B6 mice (133). In conclusion, abnormal intestinal flora may contribute to the disease development in SLE through AhR pathways.

Although both gut microbiota and AhR are closely associated with the development of SLE, the interaction between the two does not seem to play a direct role in SLE development at present.

#### 5.1.2 Ultraviolet affects the development of SLE through AhR pathways

Ultraviolet(UV) irradiation activates the AhR and promotes the production of several AhR ligands, such as FIZC (26). Among the environmental factors affecting the development of SLE, UVB is an important item. Ultraviolet B (UVB) can suppress DNA methyltransferase 1 (DNMT1) activity in CD4+ T cells of SLE patients and induce CD4+ T cells methylation-sensitive gene hypomethylation, thus exacerbating SLE. A study by Zhouwei Wu et al. confirmed that UVB can suppress SIRT1 expression through activation of AhR and subsequently suppress CD4+ T cells in SLE patients of DNMT1 activity (134). Propranolol, a potential lupus-inducing drug, induced stronger AhR activation in the PBMCs of SLE patients than in the control group, and signs of AhR activation were also shown in skin tissues related to lesion expression. Interestingly, its AhR agonist activity was increased by UVB exposure (135).

#### 5.1.3 Environmental toxicants affect the development of SLE through AhR pathways

The sources of environmental toxins are very broad and can originate from vehicle exhaust, combustion, and industrial emissions, and the main components include PAHs, dioxins, and TCDD. A large body of evidence has demonstrated that environmental pollutants affect the development of SLE. Experiments by O’Driscoll CA et al. demonstrated that different doses of air particulate extract (SRM1650b PM, SRM2975 PM) affect the differentiation of Th17 and Treg *in vitro*, thus affecting the autoimmune response, and this regulatory effect depends on AhR (136). Long-term exposure to high levels of PCBs increased SLE morbidity and mortality in women in a long-term follow-up study in a Taiwanese population (137). However, contradictory results have been reported for different AhR ligands, Li J et al. demonstrated the immunosuppressive effect of TCDD on murine SLE (138), while Amjad Mustafa et al. reported that in lupus-like autoimmune SNF(1) mice, prenatal TCDD leads to an active postnatal immune response and exacerbates lupus-like responses (139).

Smoking is an important environmental factor, the main components of cigarette smoke are nicotine, PAHs, heterocyclic compounds, heavy metal elements, etc. Numerous studies have demonstrated that previous and current smoking increases the risk of SLE and exacerbates disease severity (140, 141). Marie Saghaeian Jazi et al. investigated the polymorphisms of AhR pathway genes in smoking-related SLE patients, in their study, xenobiotic-metabolizing genes CYP1A1 and AhRR are associated with xenobiotic susceptibility and disease severity in SLE. There was an association between polymorphisms in AhRR and CYP1A1 and SLE severity only in smokers, suggesting that smoking exposure requires significant effects of xenobiotic-metabolizing genes. Smoking is a definite environmental risk factor associated with the development of SLE (142), and the components of smoke include some AhR ligands (such as dioxins and dioxin-like compounds) that may be able to trigger the AhR/AhRR/CYP1A1/B1 axis (143).

### 5.2 AhR influences the development of SLE by modulating estrogen signaling

In the previous section on AhR signaling pathway we have described the non-genomic pathway of the AhR, which involves the ER. AhR and ER are both ligand-activated transcription factors that function in the nucleus and are involved in many physiological processes, including effects on endogenous estrogen metabolism and proteasomal degradation. The physiological roles of these two nuclear receptors and the complex crosstalk between their signaling pathways have potential implications for the development of certain diseases, including SLE, which is known to be more prevalent in women and may be attributed to the effects of estrogen on the immune system. Estrogen activates the ER and acts through two different receptors, ERα and ERβ. The effects of estrogen in SLE are complex and are mainly mediated by ERα (144, 145). It has been demonstrated that women during pregnancy (146) and taking postmenopausal estrogen therapy are at increased risk of SLE (147), whereas there is a little effect between oral contraceptives and the development of SLE (144) and no increase in the risk of exacerbations in women with stable SLE (148). Notably, high levels of estrogen cannot be simply assumed to be associated with SLE. S S Shabanova et al. studied 94 untreated women with SLE, 25% of whom had reduced estrogen levels (149).

**Figure 3:**
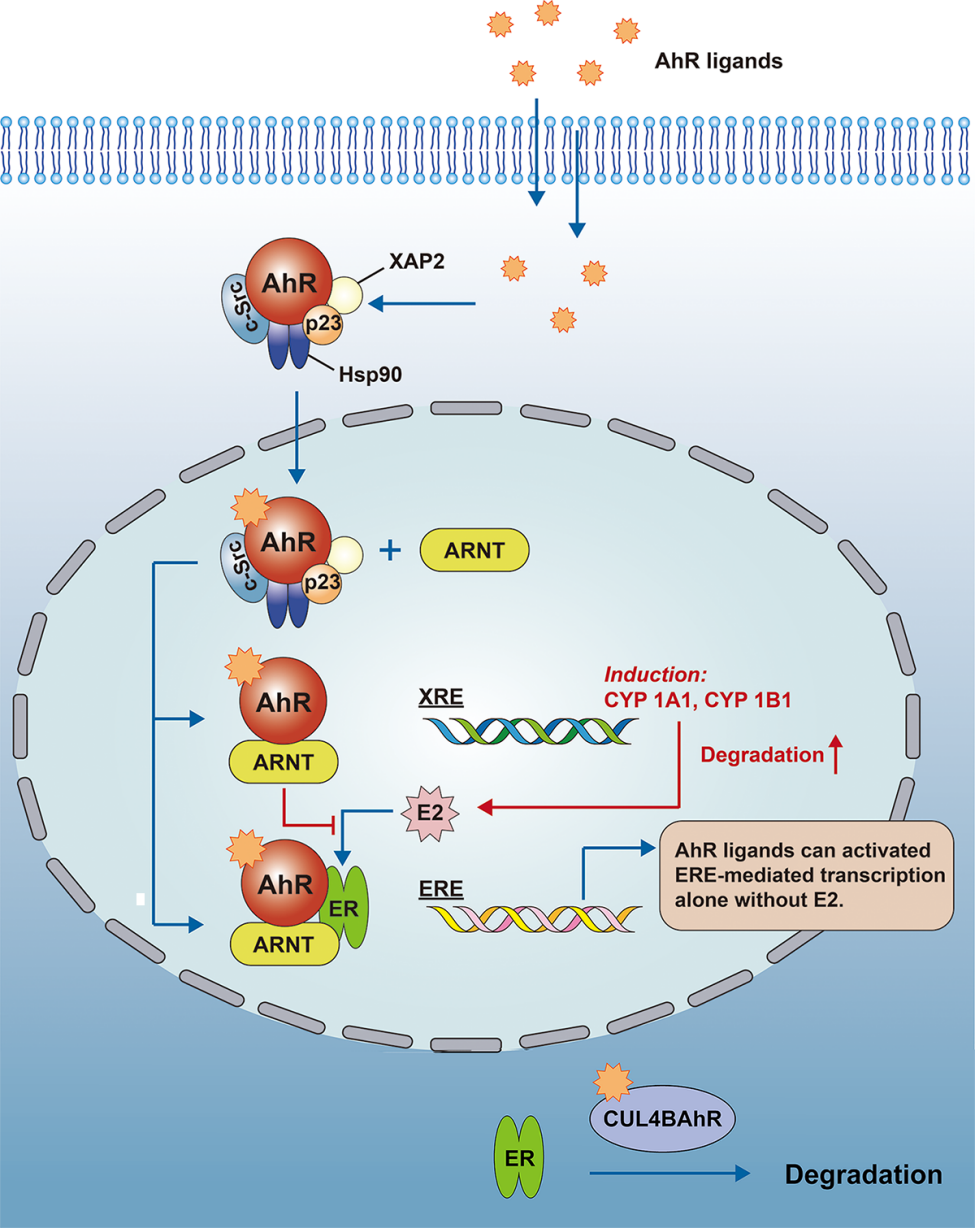
AhR regulates estrogen signaling. The AhR-ARNT complex would interact directly with the ER and bind to the ERE to activate transcription, while the AhR-ARNT complex also inhibits E2 binding to the ER. At the same time, AhR can function as an E3 ubiquitin ligase to promote the degradation of the sex hormone receptor (CUL4BAhR is a complex that consists of AhR, ARNT, CUL4B, TBL3, DDB1 and Rbx1, the AhR plays a role as an adapter for specific substrates).AhR, Aryl hydrocarbon receptor; ARNT, Aryl hydrocarbon receptor nuclear transport protein; XRE, xenobiotic response element; ERE, estrogen response element; CYP, Cytochrome P450; E2, estradiol; DDB1, damage-specific DNA binding protein 1; TBL3, transducin beta-like protein 3; Rbx1, ring-box 1.

There are multiple associations between AhR and estrogen; the AhR ligand TCDD leads to increased expression of CYP, which accelerates the metabolic degradation of estrogen (150); B Astroff et al. demonstrated that TCDD may produce anti-estrogenic effects by activating AhR (151). Whereas ARNT can also regulate estrogen signaling, ARNT can act as an estrogen receptor modulator alone, and in the presence of E2, ARNT is recruited to the estrogen response promoter, leading to an increase in ER transcription by an unknown mechanism (152). In another aspect, Ohtake et al. reported that the AhR-ARNT complex would interact directly with the estrogen receptor and bind to the estrogen response element (ERE) to activate transcription, while the AhR-ARNT complex also inhibits E2 binding to the ER, so that AhR also mediates some of the adverse estrogen-related effects (153). Meanwhile, AhR can function as an E3 ubiquitin ligase and promote ER degradation (154) (see **Figure 3**).

Based on all the above evidence, it is reasonable to speculate that AhR can influence the occurrence and development of human SLE by regulating estrogen signaling.

### 5.3 AhR affects immune cells in SLE

Many immune cells are involved in AhR-induced SLE pathogenesis. According to the previous section, Th17 and Treg cells are key players in the immune response. The homeostasis of the Th17/Treg cell is critical to the pathogenesis of SLE (155), it has been shown that the proportion of Th17 cells is elevated and Treg cells are reduced in SLE patients (156, 157). In a case-control study, Haitao Yu et al. examined the relationship between the ratio of AhR in Th17 cells and the ratio of AhR in Treg cells and SLE skin lesions in SLE patients (158). In the analysis of PBMCs from SLE patients, AhR expression was more than three-fold higher than that in healthy controls, and patients in the high AhR ratio group had more extensive lesions and more decreased C3 levels compared to the low AhR ratio group.

Fcγ receptor IIb (FcgRIIb)-deficient (FcgRIIb-/-) mice can develop lupus-like disease and some environmental contaminants can activate AhR and cause amplify inflammatory responses (159). Kanyarat Udompornpitak et al (160) used the AhR agonist 1,4-chrysenequinone (1,4-CQ) to produce a strong response in macrophages of FcgRIIb-/- mice compared to macrophages of wild-type mice, and these activations led to a more severe inflammatory response, causing FcgRIIb-/- mice to develop lupus-like features (161).

### 5.4 AhR pathway affects SLE development in a ligand-dependent manner

As mentioned previously, a variety of environmental toxicants are ligands for the aryl hydrocarbon receptor and exposure to these environmental toxicants can exacerbate SLE. Other exogenous ligands, such as quercetin, curcumin and resveratrol as dietary components can bind to AhR and improve SLE symptoms (162–164). Leflunomide as an agonist of AhR suppresses the immune response and treats a variety of diseases, including SLE (165, 166).

As one of the endogenous ligands, I3C has been shown to be a beneficial factor in regulating the inflammatory response and cytokine expression in SLE. Saeed Mohammadi et al. showed (167) that I3C-mediated activation of AhR significantly downregulated the overexpression of inflammatory cytokines and also had an immunomodulatory effect on macrophages in SLE patients. Kyn, as an established endogenous ligand of AhR, can exert anti-inflammatory and immunomodulatory effects through activation of AhR (168) and mTOR (62), and we have previously mentioned that Kyn has a definite effect on SLE. In a quantitative metabolomic analysis of peripheral blood lymphocytes (PBL) from SLE patients, Andras Perl et al. found that Kyn was one of the most increased metabolites (62). Treatment with N-acetylcysteine (NAC) blocked mTOR (which is extensively linked with AhR, details in **5.5**) in T lymphocytes and significantly reduced Kyn levels in patients, suggesting a therapeutic role in SLE (169).

It is important to note that we cannot simply attribute the effect of ligand activation of AhR on SLE to the agonist/antagonist activity of the ligand. For example, while resveratrol is an AhR antagonist and leflunomide is an AhR agonist, they both have a therapeutic effect on SLE. Just as two different AhR agonists, TCDD and FICZ, have opposite effects on EAE development (76). Since each ligand may have different effects on the disease, it is important to determine the detailed mechanisms by which each ligand affects AhR signaling.

### 5.5 AhR influences the development of SLE through multiple signaling pathways

**Figure 4:**
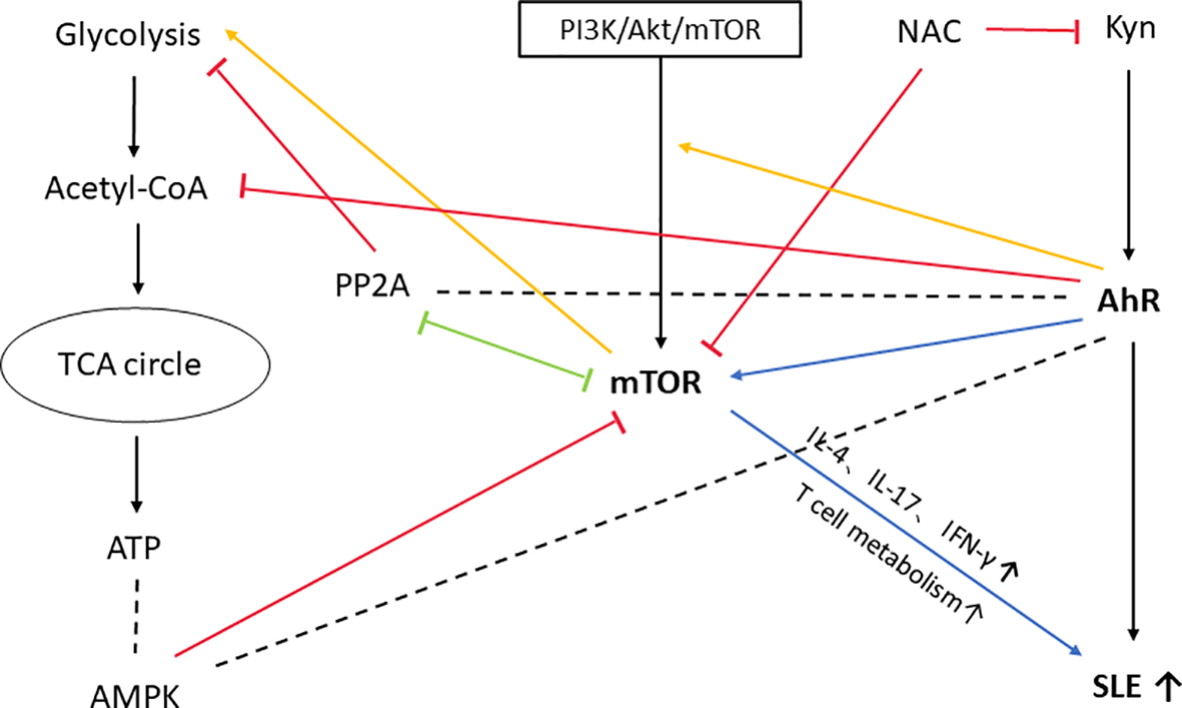
mTOR, an important molecule involved in the development of SLE by AhR. In this figure, we only show the possible association between themTOR and AhR pathways and their effects on SLE. AhR can reduce acetyl coenzyme A levels and thus have an effect onmTORC1-induced glycolysis. mTORC1-induced glycolysis is also inhibited by PP2A, which has a negative crosstalk with mTORC1 and is a regulator of AhR. AMP-activated protein kinase (AMPK) is regulated by ATP/ADP or AMP/ATP ratio in the cell. Activated AMPK inhibits the activity of mTORC1 (179). AMPK is also regulated by AhR-driven degradation of Synphilin-1, which can reduce AMPK production (180). So PP2A and AMPK may be importantmolecules linking mTOR to AhR. PI3K/Akt/mTOR pathway is one of the upstream pathways regulating mTOR, and AhR enhances the activation of PI3K/Akt/mTOR signaling pathway, thus promoting cell survival. Kyn itself has a powerful pro-inflammatory effect and can induce SLE development by activating AhR. Moreover, Kyn activation ofmTOR is also involved in the development of SLE, a process accompanied by production of cytokines such as IL-4, IL-17, IFN-γ, and increased T-cell metabolism. Treatment with NAC inhibits mTOR activation and significantly reduces Kyn levels in SLE patients. PP2A, protein phosphatase 2A; AMPK, AMP-activated protein kinase; PI3K, phosphatidylinositol-3-kinase; Akt, autologous tumor killing; NAC, N-acetylcysteine.

In the previous description, we mentioned the mTOR signaling pathway, which is closely related to the pathogenesis of SLE (170). Moreover, the AhR and mTOR pathways have been extensively connected and AhR activation can stimulate mTOR pathway activation (171). Fangyi Shi et al. found that AhR enhanced activation of the PI3K/Akt/mTOR signaling pathway, thereby promoting cell survival (172). According to George Anderson et al (171), the link between mTOR and AhR activation is also widely present *via* metabolic pathways in the tumor microenvironment, the backbone of which is built up by oxidative phosphorylation and glycolysis regulated through the acetyl coenzyme A and melatonin pathways. The AhR can attenuate acetyl-CoA levels, with consequences for mTORC1 induction of amino acid transporter(LAT1) and glycolysis. Protein phosphatase 2A(PP2A) is recognised as a regulator of the tumour microenvironment (173), which can inhibit mTORC1-induced LAT1 and glycolysis, and there is a negative crosstalk between PP2A and mTORC1 (174). PP2A is also a regulator of the AhR (175). The involvement of mTOR activation in SLE was first mentioned by Fernandez DR et al (176). Furthermore, mTOR is involved in the proliferation and differentiation of immune cells and the production of inflammatory cytokines in SLE, and the AhR signaling pathway may be involved. The effect of mTOR blockade on cytokine production in SLE patients has been proved (177, 178). Tryptophan and its metabolites increase T cell metabolism and mTOR activation, and Kyn promotes IFN-γ production, all of which are associated with the development of lupus in mice (132). Overall, the pro-inflammatory effects of AhR stimulation by Kyn and the activation of mTOR by Kyn play an important role in the pathogenesis of SLE, there is likely to be a mutually reinforcing relationship between the two. For more links between mTOR, AhR and SLE please see **Figure 4**.

Kyn itself has a powerful pro-inflammatory effect and can induce SLE development by activating AhR. Moreover, Kyn activation of mTOR is also involved in the development of SLE, a process accompanied by production of cytokines such as IL-4, IL-17, IFN-γ, and increased T-cell metabolism. Treatment with NAC inhibits mTOR activation and significantly reduces Kyn levels in SLE patients.

Programmed death (PD)-1 signaling pathway contributes to the development of SLE. According to a recent report by Colleen S. Curran et al. (181), the PD-1 signaling cascade is regulated by the Toll-like receptor (TLR) pathway and the type I interferon (IFN) pathway by activating NF-κB and/or STAT1. Tyrosine kinase receptors (TAM) are the main regulators of these signals. In contrast, dysregulated cellular signaling in SLE can identify pathways involved in the control of PD-1 responses. The other two pathways mentioned before are also two key pathways affecting SLE pathogenesis (182, 183). FICZ, Kyn, and other AhR ligands increase or inhibit PD-1 and PD-L1 expression on cell surfaces, and as previously described, AhR is also expressed in various cells (such as Th17, Treg) that exhibit crosstalk with NF-κB and STAT1.

Also associated with SLE is the germinal center kinase-like kinase (GLK) pathway, which produces the cytokine IL-17A. The frequency of T cell overexpression of GLK shows a positive correlation with disease severity in SLE patients. Chuang HC and other investigators have found that in animal experiments, GLK signaling stimulates IL-17A production in mice Th17 cells by inducing the formation of the AhR-retinoic-acid-receptor-related orphan nuclear receptor γt (ROR-γt) complex, This process is highly selective and promotes autoimmunity (184). Their recent findings suggest (185) that the GLK-induced AhR-ROR-γt complex in Th17 may serve as a marker of IL-17A-mediated autoimmune disease and could be a new therapeutic target for human SLE and several diseases.

## 6 Summary

AhR is known as an environmental receptor, it’s a cytoplasmic receptor and transcription factor that is activated by binding to the corresponding ligands, and it transmits relevant information by binding to DNA, thus activating the transcription of various genes. There are many other interesting receptors similar to AhR in the human body, such as the retinoic acid-inducible gene I (RIG-I)-like receptor (RLR) and NOD-like receptor (NLR). RLR is a key sensor of viral infection that mediates transcriptional induction of type I interferons and other genes that co-establish antiviral host responses, both viral and host-derived RNAs can activate RLR (186). NLR is an intracellular innate immune sensor that sense intracellular microbial and non-microbial danger signals and form large cytoplasmic complexes called inflammasomes that link the sensing of microbial products and metabolic stress to the proteolytic activation of the proinflammatory cytokines IL-1beta and IL-18 (187). However, AhR differs from them in that it possesses ligands of diverse origin and is associated with much more different internal and external environmental factors, so AhR may have a broader role. It is necessary to study such receptors with sensor roles because they are often an important part of multiple pathophysiological responses. For this review, we focused on the role of AhR in the development of autoimmune diseases, mainly SLE, in order to provide clues for more effective and targeted prevention and treatment methods.

AhR contributes to many autoimmune diseases, and this review focuses on outlining the role of AhR in the development of different subtypes of immune cells and SLE. AhR ligands are mainly of exogenous and endogenous origin, but their effects are complex, with ligand-, dose- and environment-specific responses. For example, small doses of FICZ promote mast cell differentiation while large doses suppress it; some ligands have different effects in different environments, such as TCDD, which promotes Th17 cell differentiation *in vitro* but suppresses it *in vivo*. Moreover, what is clear about AhR ligands and autoimmunity is that most AhR ligands cause immunosuppression and improve autoimmune disease, while others exacerbate disease (especially environmental toxins, but which have both immunosuppressive and stimulatory effects). Different AhR ligands alter the balance between regulatory T cells and the outcome of autoimmune diseases. The specific mechanisms responsible for these complex, even contradictory effects may be as follows: First, the route of exposure and the degree and duration of AhR activation contribute to altering the effects of AhR ligands on autoimmunity; second, the lower affinity of AhR expressed in humans compared to animal models prevents the production of sufficient amounts of pro-inflammatory cytokines *in vivo*; and finally, AhR as a member of multiple signaling can be regulated by many different genes, substances, or environments, and subtle differences in cascade effects may make AhR act differently.

From the results of current studies on AhR and SLE, some experiments have demonstrated that AhR activation can exacerbate the development of SLE, such as the action of UV, environmental toxins, estrogen, etc. with AhR. However, there is still much evidence that ligand activation of AhR improves SLE symptoms, such as I3C and resveratrol, which suggests that AhR is a promising therapeutic target for autoimmune diseases. However, there are some interesting results, such as evidence that AhR activation can have different effects on lupus in mice and humans: Rahul Shinde et al. reported that blocking AhR activity in mice enhanced autoimmune responses and that activation of AhR improved lupus-like disease (45). However, increased AhR activity was found in human SLE patients (188, 189). Although the authors have not yet elucidated the reason, we speculate that this also seems to be related to the different affinities of AhR expressed in humans and mice as mentioned previously.

**Figure 5:**
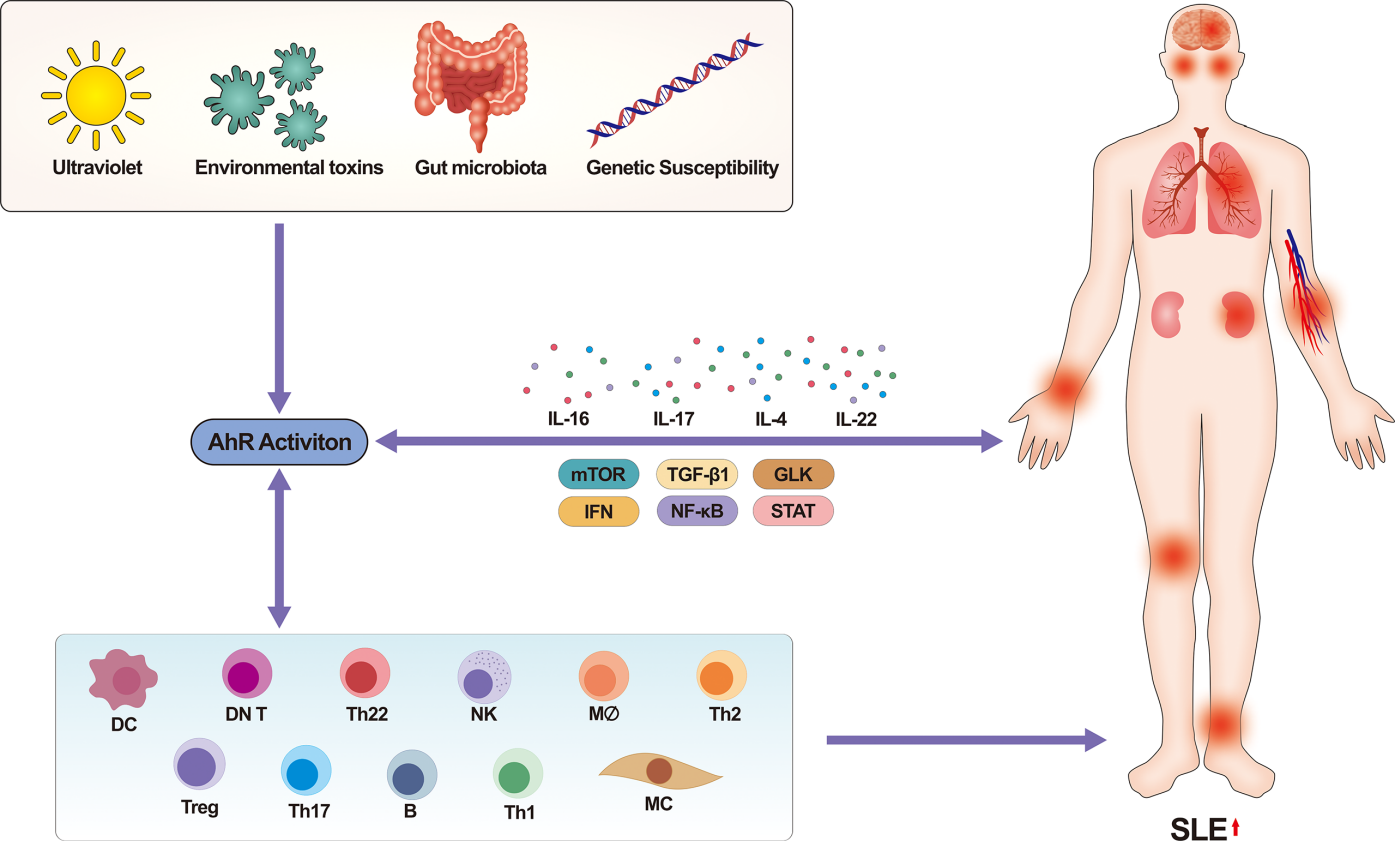
Activation of AhR by multiple factors promotes the occurrence and development of human SLE. On the one hand, factors such as ultraviolet, environmental toxins, and gut microbiota will mostly affect the binding of AhR to ligands. AhR regulates the pathophysiological functions of multiple immune cells in a ligand-specific manner, which in turn affects SLE. Meanwhile, many signaling pathways that regulate disease (e.g., mTOR, PD-1, NF-κB, STAT, TGF-β1, GLK signaling pathway, etc.) also involve activation of AhR, which promotes the development of SLE with the involvement of multiple cytokines. On the other hand, immune cells also secrete various pro-inflammatory substances and cytokines that influence the activation of certain signaling pathways. In conclusion, multiple pathways together contribute to the development of SLE through AhR. mTOR, mechanistic target of rapamycin, IFN, interferon, STAT, signal transducer and activator of transcription, TGF, transforming growth factor, GLK, germinal center kinase-like kinase, SLE, systemic lupus erythematosus.

A variety of factors such as gut microbiota, environmental toxins (mainly PAHs, dioxins), and genetic susceptibility can influence SLE development through the AhR pathway. In addition to being involved in regulation in a ligand-specific manner, AhR itself is associated with a variety of intercellular signaling, and these factors together influence the development of SLE (**Figure 5** summarizes the effect of multiple factors on the occurrence and development of SLE after activation of AhR). Therefore, in-depth research of the mechanisms of AhR in the development of SLE will hopefully inspire new ideas and targets for the prevention or treatment of SLE. However, we still face many problems: firstly, a further epidemiological investigation is needed to verify the relationship between AhR ligands and SLE; secondly, the number of studies related to AhR and SLE is small and the studies don't far enough, many mechanisms are still unexplored. At present, various diagnostic tools for SLE are developing rapidly, such as the application of bioinformatics, proteomics, biomechanics and functional analysis. Although the road to a brighter future is bumpy and winding, and it will take a long time to figure out the way forward, we believe that more AhR-related researches will also shine in SLE in the near future.

## Author contributions

JW wrote the manuscript and prepared the figures. HJ, MZ reviewed the manuscript. TP, ZL participated in reference search and discussion of the manuscript. All authors contributed to the article and approved the submitted version.

## Funding

This study was supported by the National Natural Science Foundation of China (No. 81874253), Excellent postdoctoral innovative talents of Hunan province in 2020 (No. 2020RC2014), Natural Science Foundation of Hunan Province China (No. 2021JJ40837). The 15th medium-term special grant of postdoctoral Science Foundation of China (2022T150742). The authors did not have financial support or benefits from commercial resources.

## Conflict of interest

The authors declare that the research was conducted in the absence of any commercial or financial relationships that could be construed as a potential conflict of interest.

## Publisher’s note

All claims expressed in this article are solely those of the authors and do not necessarily represent those of their affiliated organizations, or those of the publisher, the editors and the reviewers. Any product that may be evaluated in this article, or claim that may be made by its manufacturer, is not guaranteed or endorsed by the publisher.
